# Priority Management of Henle Trunk in Cranial-to-Caudal Approach for Laparoscopic Right Hemicolon Cancer Surgery

**DOI:** 10.3389/fsurg.2022.883973

**Published:** 2022-04-26

**Authors:** Yao Yang, Xiaohua Jiang, Zhuqing Zhou, Bing Lu, Zhe Zhu, Qixing Jiang, Fang Ji, Chuangang Fu

**Affiliations:** ^1^Department of Colorectal Surgery, Shanghai East Hospital, School of Medicine, Tongji University, Shanghai, China; ^2^Department of General Surgery, Shanghai East Hospital, School of Medicine, Tongji University, Shanghai, China

**Keywords:** laparoscopic surgery, radical right hemicolectomy, Henle trunk, intraoperative blood loss, operation time

## Abstract

This study aimed to compare the short-term clinical efficacy between prior and traditional approach of Henle trunk in laparoscopic right hemicolectomy (LRH) for right colon cancer. A total of 161 patients underwent LRH for right colon cancer between June 2018 and December 2020 by the same group of physicians. The prior approach of Henle trunk (priority group) was used in 82 patients and traditional approach in 79 (traditional group). The demographics and clinicopathological characteristics were recorded and retrospectively analyzed. As compared to the traditional group, the mean blood loss reduced significantly [73.84 ± 17.31 mL vs. 83.42 ± 30.16 mL; *P* = 0.001], the operation time was markedly shorter [151.35 ± 6.75 min vs. 159.13 ± 18.85 min; *P* = 0.014], and the intraoperative vascular injury rate was significantly lower [6.1% (5/82). vs. 17.7% (14/79); *P* = 0.022]. There were no significant differences in the postoperative complications, first exhaust time, first defecation time, drainage time, postoperative hospital stay, quality evaluation of surgical specimens and pathological findings between two groups. Our study shows that the priority management of Henle trunk in the LRH for right colon cancer is a safe and feasible procedure with less blood loss, shorter operation time and lower intraoperative vascular injury rate.

## Introduction

Laparoscopic technology has experienced rapid development in the past 30 years. Laparoscopic radical right hemicolectomy, a modality recommended by the National Comprehensive Cancer Network (NCCN) guideline and other guidelines, has gain popularity in clinical practice (1, 2). Proper dissection of the Henle trunk during the laparoscopic radical right hemicolectomy for colon cancer is important for reducing surgical complications. Henle trunk, also known as gastrocolic trunk (GCT), was discovered in 1868 by Dr. Henle of Germany who found that the superior right colic vein (SRCV) and right gastroepiploic vein (RGEV) converged to form a venous trunk (3). Other studies have also revealed that it also collects anterior superior pancreaticoduodenal vein (ASPDV) along its course (4), forming the classic anatomy of Henle trunk. As the anatomy of right colon is more complex, vascular variation is more common in the right colon than in the left colon and, in particular, the variation of Henle trunk is frequently encountered in clinical practice. Thus, the identification and confirmation of this structure have been a challenge, and any mistake may cause accidental vascular injury and intraoperative bleeding (5). Therefore, the strategies for Henle trunk management (for example, how to optimize surgical approach and technique) have become a focus in studies. The middle approach is a classic surgical approach for LRH for colon cancer. It basically traces along superior mesenteric vein and dissects and exposes vessels in a top-up way toward Henle trunk where management is given. Some studies have also reported the cephalic approach by entering the omental sac, dissecting transverse mesocolon, identifying right gastroepiploic vein as the marker, and tracing along the vessels in a top-down way while giving priority to exposing and managing Henle trunk and its branches (6). The short-term outcome of these two techniques in the laparoscopic right hemi-colectomy (LRH) for right colon cancer has not been reported yet. In the present retrospective study, the clinical data of 161 patients who underwent LRH for right colon cancer between July 2018 to December 2020 were analyzed, and the priority and traditional techniques for Henle trunk in this procedure were compared in terms of safety and feasibility.

## Methods

### Clinical Data

Patients who received LRH for right colon cancer by the same group of physicians in the Department of Colorectal Surgery of Shanghai East Hospital, Tongji University between July 2018 and December 2020 were included into present study by reviewing surgery video recordings. A total of 161 patients were included, including 82 patients who had priority management of Henle trunk (priority group) and 79 patients who received traditional approach (traditional group). This study was approved by the Ethical Review Board of Shanghai East Hospital. Informed consent from patients was waived due to the retrospective nature of this study. Table 1 summarizes the baseline characteristics of patients in two groups.

**Table 1:** Baseline characteristics of patients in the priority group and traditional group.

**Variables**	**Priority group** **(*n* = 82)**	**Traditional group** **(*n* = 79)**	**Statistics**	***P***
Gender (male/female, *n*)	41/41	39/40	*x*^2^ = 0.006	0.936
Age (years)	64.32 ± 12.24	66.68 ± 11.96	*t* = 1.240	0.217
Body mass index (kg/m^2^)	23.63 ± 2.89	22.82 ± 3.17	*t* = 1.698	0.092
Tumor dimension (cm)	4.49 ± 1.57	4.36 ± 1.62	*t* = 0.515	0.607
Tumor location [*n* (%)]			*x*^2^ = 2.214	0.330
Ileocecal	27 (32.9)	22 (27.8)		
Ascending colon	30 (36.6)	38 (48.1)		
Hepatic flexure	25 (30.5)	19 (24.1)		
T-stage [*n* (%)]			χ^2^ = 0.329	0.848
T1	3 (3.7)	3 (3.8)		
T2	9 (10.9)	11 (13.9)		
T3	70 (85.4)	65 (82.3)		
N-stage [*n* (%)]			χ^2^ = 1.053	0.305
Positive	44 (53.7)	36 (45.6)		
Negative	38 (46.3)	43 (54.4)		
Tumor clinical staging			χ^2^ = 2.986	0.225
[*n* (%)]				
I	1 (1.2)	3 (3.8)		
II	7 (8.6)	12 (15.2)		
III	74 (90.2)	64 (81.0)		

### Inclusion and Exclusion Criteria

Inclusion criteria: (1) solitary colon malignant tumor located in the ileocecal, ascending colon, and hepatic flexure was confirmed by preoperative electronic colonoscopy and pathological examination. (2) Laparoscopic radical right hemicolectomy was performed with cranial-to-caudal approach (7) or just middle approach.

Exclusion criteria: (1) The tumor diameter was > 10 cm on preoperative and intraoperative assessment; (2) there were peritoneal cavity implantation or remote metastasis of cancer cells; (3) there were severe comorbidities of cardiopulmonary or other organs; (4) patients had a prior history of colorectal surgery.

### Surgical Procedures

All procedures followed the principle of complete mesocolic excision (CME) (8) and D3 lymph node dissection was employed. In the priority group, cranial-to-caudal approach was used for LRH, and just middle approach in the traditional group.

#### Portal Configuration

**Figure 1:**
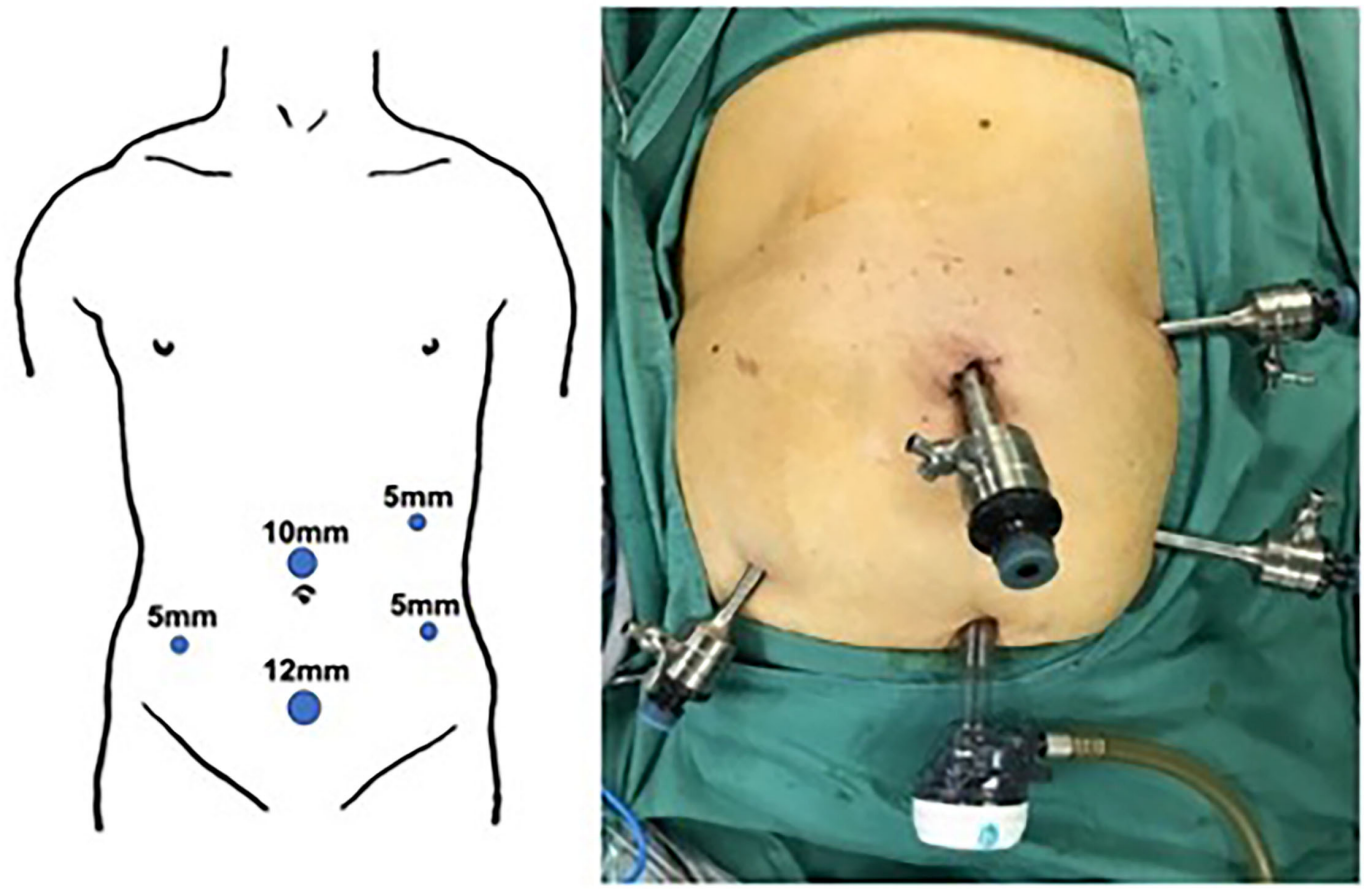
Illustrative photo of 5 portals.

A 10-mm trocar was placed on the upper edge of the umbilicus for observation. The remaining 4 trocars included: 5-mm trocar placed on the left anterior axillary line and 5 cm above and 5 cm below the umbilicus; 5-mm trocar placed on the right anterior axillary line, 5 cm below the umbilicus; 12-mm trocar placed about 8 cm below the umbilicus (Figure 1).

The laparoscope was located on the right front of the patient, while the surgeon stood on the left side of the patient, the laparoscope holder stood on the left side of the surgeon, and the assistant stood between the legs of the patient.

#### Priority Group

**Figure 2:**
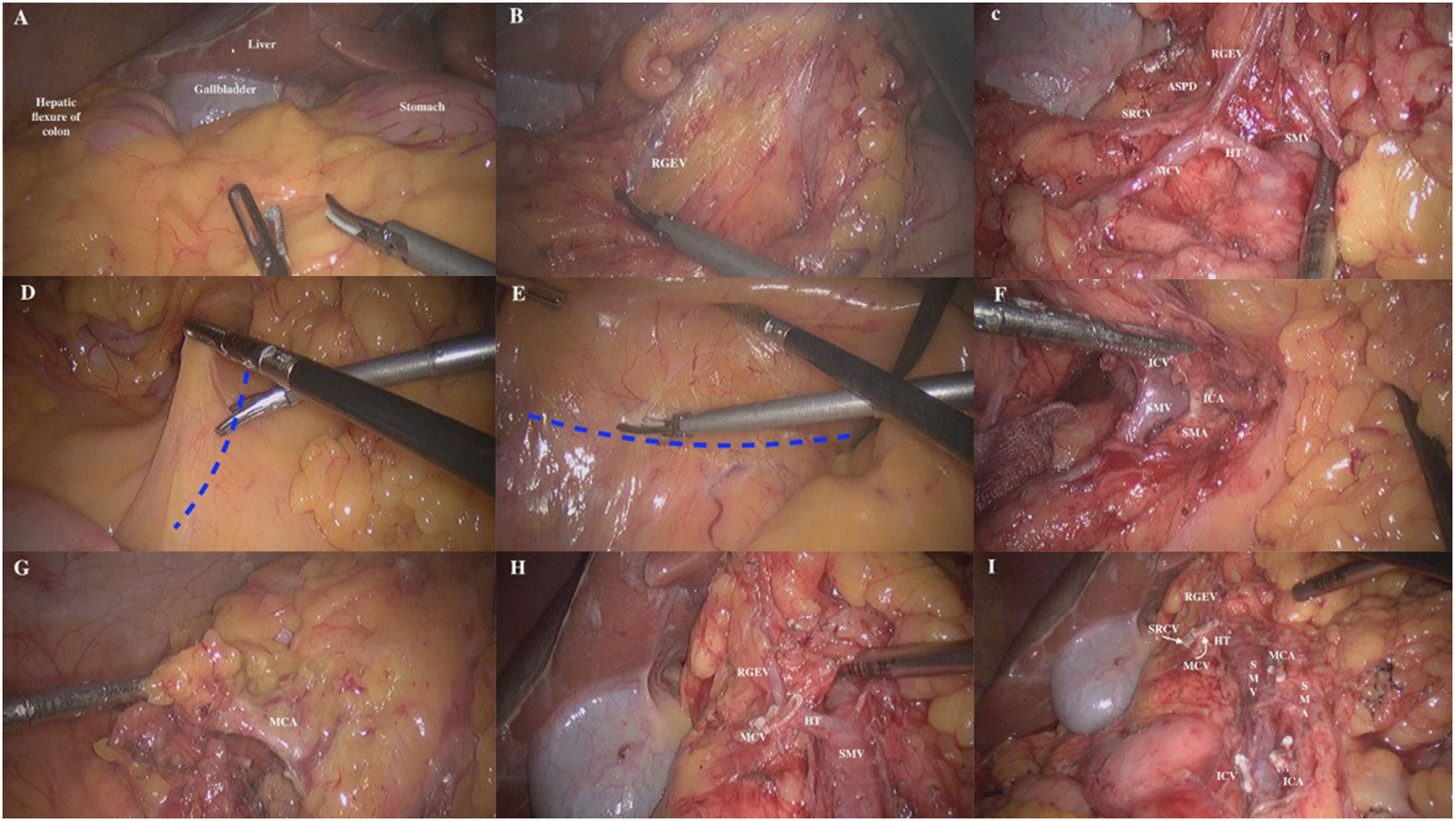
The steps of priority management of the Henle trunk in cranial-to-caudal approach. **(A)** The gastric-colon ligament was incised along the lateral edge of gastroepiploic vessel arch. **(B)** The RGEV was exposed and dissected at anterior lobe of the transverse mesocolon. *RGEV* right gastroepiploic vein. **(C)** Along the RGEV, the SRCV was dissected and exposed. **(D)** The projection of SMV served as the trajectory of incision and was marked. **(E)** The endoscope entered the fusion fascia space from the lower portion of the horizontal part of the duodenum. **(F)** The SMV was exposed to the right side of SMA, and then the SMA, SMV, and their branches were dissected. **(G)** The branches of SMA were dissected upward along the left side of this vessel. The MCA was ligated and incised. *MCA* middle colon artery. **(H)** The MCV was ligated and incised. **(I)** The regional lymph node dissection was performed.

(1) Patient's head was elevated by 30° the peritoneal cavity was fully explored. The projection point of the ligamentum teres hepatis on the omentum served as the start point, and the omentum was retracted, the gastric-colon ligament was incised along the lateral edge of gastroepiploic vessel arch until to the right side of the junction of transverse mesocolon and stomach (Figure 2A). The anterior lobe of the transverse mesocolon, anterior part of pancreas and descending part and horizontal part of the duodenum were exposed. The anterior lobe of the transverse mesocolon was incised from the lower edge of the pancreas at the neck of the pancreas by entering the fusion fascia space. At this point, the right gastroepiploic vein (RGEV) was identified (Figure 2B). Along the RGEV, this served as a land marker, the superior right colon vein (SRCV) was dissected and exposed. The superior anterior pancreaticoduodenal vein (ASPDV), middle colon vein (MCV) and Henle trunk were gradually separated and exposed, and the partial superior mesenteric vein (SMV) was exposed (Figure 2C). Then, the SRCV was ligated at the confluence of the RGEV. The management of MCV was left for the middle approach. For tumors at the hepatic flexure, the middle colon vessels were ligated and incised at the root level. (2) The patient's head was lowered by 30° and tilted by 15° to the left. The omentum and ileum were pushed to the upper left abdomen. The projection of superior mesenteric vein (SMV) served as the trajectory of incision and was marked (Figure 2D). The assistant lifted the appendix and the terminal mesoileum to the ventral side, fully exposing the “yellow-white junction line” between the root of terminal mesoileum and the retro peritoneum. The fusion fascia space was entered from the lower portion of the horizontal part of the duodenum (Figure 2E). The fusion fascia space was fully extended to the reproductive vessels on the right, till the left edge of the SMV medically, and then extended to the cranial side. Finally, the upper free space was joined to complete the dissection of right mesocolon. (3) The transverse mesocolon was lifted up vertically to expose and identify the projection of SMV on the mesentery surface. The course of SMV and ileocolonic vessels were confirmed. The ileocolonic vessels was lifted up, the mesentery below it was incised, and dissected upwards to expose SMV to the right side of SMA. The SMA, SMV as well as their branches were further dissected. The ileocolonic artery and vein were ligated and incised at the root level (Figure 2F). Subsequently, the branches of SMA were dissected upward along the left side of this vessel. The middle colon artery (MCA) was ligated and incised (Figure 2G), and the MCV was ligated and divided (Figure 2H). Then, the regional lymph node dissection was performed (Figure 2I). (4) The insufflation was stopped, the observation portal was extended above the umbilicus, the colon was brought out of the abdominal cavity and the tumor was resected. Then, an end-to-end or end-to-side anastomosis was made between the ileum and transverse colon outside the abdominal cavity. Insufflation was re-established, the abdominal cavity was irrigated, and the drainage tube was placed, followed by wound closure.

#### Traditional Group

Middle approach was adopted (9). The mesentery was incised along the projection of superior mesenteric vessel. First, the ileocolonic vessels were dissected and managed. The right and middle colon vessels were dissected in a top-up manner and then ligated and incised. Finally, the Henle trunk and its branches were dissected and managed, while RGEV and ASPDV were retained. The gastrocolonic ligament were dissected and incised from the cranial side lateral to gastroepiploic arch. For tumors at the hepatic flexure, branches of the right gastroepiploic vessel should be incised. The retroperitoneum was incised along the lateral side of the colon from the iliac fossa to the hepatic flexure, and the ascending colon was dissected from the posterior abdominal wall. The tumor was resected and an end-to-end or end-to-side anastomosis was made between the ileum and transverse colon outside the abdominal cavity.

### Criteria for Evaluation

1. General characteristics: the general characteristics (such as gender, age, body mass index [BMI], tumor dimension, tumor location and tumor clinical staging) were recorded.
2. Evaluation of safety and short-term outcome: The operation time, intraoperative blood loss, intraoperative vessel injury, intraoperative blood transfusion, postoperative complication rate, first postoperative exhausting time, first postoperative defecation time, postoperative drainage time, postoperative hospital stay and quality evaluation of surgical resection specimens were recorded. The quality evaluation of colon specimen was evaluated based on intraoperative photos by the grading system of West et al. (10). Grade A is at muscular is propria level; Grade B is at internal mesocolon level. Grade C is at mesocolonic level (smooth and complete resection of the mesocolon). Grade D is mesocolon level with high feeder artery ligation close to the aorta. The operation time is defined as the time from the initial insufflation to the end of operation. The intraoperative bleeding is defined as intraoperative visible vascular bleeding. The blood loss refers to the volume of blood after deducting the abdominal effusion before the abdominal cavity flushing plus the blood in the endoscopic gauze (nominally 10 mL as per gauze). Intraoperative vascular injury is defined as the injury to the branches of Henle trunk (including right gastroepiploic vein, right colon vein, right accessory colon vein, middle colon vein, and superior pancreaticoduodenal vein) (11).
3. Postoperative pathological examination: pathological classification, total number of lymph nodes dissected, number of malignant lymph nodes dissected, surgical margin status, and pathological staging.

### Statistical Analysis

SPSS version 20.0 was used for statistical analysis. The quantitative data are expressed as mean ± standard deviation ($\bar{x}$±*s*) and compared with independent sample *t*-test between two groups. The qualitative data are expressed as frequency and percentage and compared with χ^2^ test or Fisher's exact test. A value of *P* < 0.05 was considered statistically significant.

## Results

### General Characteristics of Patients

There were no marked differences in the age, gender, BMI, tumor location, and clinical staging of colon cancer between two groups (all *P* > 0.05). In these patients, 82 underwent LRH for colon cancer via cranial-caudal approach and 79 received radical right hemicolectomy for colon cancer via middle approach. All the patients had R0 resection based on the pathological examination of surgical specimen margin. None experienced conversion to laparotomy. Death was not reported during the perioperative period.

### Safety and Short-Term Outcomes

The intraoperative blood loss (73.84 ± 17.31 mL vs. 83.42 ± 30.16 mL, *t* = 2.483; *P* = 0.001), and operation time (151.35 ± 6.75 min vs. 159.13 ± 18.85 min, *t* = 3.509; *P* = 0.014) significantly reduced in the priority group. Intraoperative vessel injury rate also reduced significantly (6.1% [5/82] vs.17.7% [14/79], χ^2^ = 5.223; *P* = 0.022) in the priority group. In the priority group, postoperative complications were observed in eight patients, including wound infection in two patients, postoperative ileas in two, abdominal effusion in one, anastomotic bleeding in 1, urinary tract infection in one, and pneumonia in one. In the traditional group, postoperative complications were noted in seven patients, including wound infection in two patients, postoperative ileus in one, abdominal effusion in one, anastomotic bleeding in two, and pneumonia in one. All three cases of anastomotic bleeding were managed via endoscopy and conservative treatment, and the remaining complications were improved after symptomatic treatment. There were no marked differences in the incidence of postoperative complications, first postoperative exhausting time, first postoperative defecation time, postoperative drainage time, postoperative hospital stay, and quality evaluation of surgical specimens between two groups (*P* > 0.05) (Table 2).

**Table 2:** Comparison of intraoperative and postoperative parameters.

**Variables**	**Priority group** **(*n* = 82)**	**Traditional group** **(*n* = 79)**	**Statistics**	***P***
**Intraoperative**				
Bleeding volume (mL)	73.84 ± 17.31	83.42 ± 30.16	*t =* 2.483	0.001
Operation time (min)	151.35 ± 6.75	159.13 ± 18.85	*t =* 3.509	0.014
Vascular injury [*n* (%)]	5 (6.1)	14 (17.7)	χ^2^ = 5.223	0.022
Intraoperative blood transfusion [*n* (%)]	2 (2.4)	4 (5.1)	χ^2^ = 0.772	0.379
**Postoperative**				
Complication [*n* (%)]	8 (9.8)	7 (8.9)	*x*^2^ = 0.038	0.845
First exhaust time (d)	3.79 ± 0.70	3.73 ± 0.78	*t =* 0.502	0.616
First defecation time (d)	5.82 ± 0.80	5.75 ± 0.71	*t =* 0.588	0.557
Postoperative draining time (d)	6.87 ± 0.78	6.68 ± 0.81	*t =* 1.454	0.148
Postoperative hospital stay (d)	11.26 ± 4.94	10.59 ± 3.57	*t =* 0.970	0.334
Quality evaluation of surgical specimen (Grade A/B/C or above)	0/2/80	0/4/75	χ^2^= 0.772	0.379

### Results of Postoperative Pathological Examination

Negative proximal and distal resection margins were observed in all the tumor specimens from two groups. There were no significant differences in the gross tumor classification, histological classification, total number of lymph node dissected, number of malignant lymph node dissected, and tumor stage between two groups (*P* > 0.05) (Table 3).

**Table 3:** Postoperative findings from pathological examination.

**Variables**	**Priority group (*n* = 82)**	**Traditional group (*n* = 79)**	**Statistic**	***P***
Morphological classification [*n* (%)]			χ^2^ = 0.767	0.681
Infiltrative	29 (35.4)	23 (29.1)		
Ulcerative	33 (40.2)	36 (45.6)		
Protuberant	20 (24.4)	20 (25.3)		
Histological classification [*n* (%)]			χ^2^ = 0.498	0.779
Adenocarcinoma	67 (81.7)	61 (77.2)		
Adenocarcinoma, partially mucinous adenocarcinoma	10 (12.2)	12 (15.2)		
Mucinous adenocarcinoma	5 (6.1)	6 (7.6)		
Number of lymph node dissected (*n*)	17.99 ± 5.22	18.39 ± 5.28	*t =* 0.489	0.626
Malignant lymph node dissected (*n*)	2.06 ± 3.50	1.77 ± 3.33	*t =* 0.537	0.592
T-stage [*n* (%)]			χ^2^ = 0.651	0.722
T1	2 (2.4)	3 (3.8)		
T2	7 (8.5)	9 (11.4)		
T3	73 (89.1)	67 (84.8)		
N-stage [*n* (%)]			χ^2^ = 1.115	0.573
N1	40 (48.8)	39 (49.4)		
N2	27 (32.9)	30 (38.0)		
N3	15 (18.3)	10 (12.6)		
Pathologic tumor staging [*n* (%)]			χ^2^ = 1.542	0.463
I	1 (1.2)	3 (3.8)		
II	7 (8.5)	9 (11.4)		
III	74 (90.3)	67 (84.8)		
IV	0 (0.0)	0 (0.0)		

## Discussion

With the concept of CME proposed by Hohenberger in 2009, the extent of resection has been further standardized for the colon cancer surgery, and the quality control of surgery is now performed more objectively. The new practice reduced the local recurrence rate of colon cancer while improving the long-term survival rate (8). When D3 lymph node dissection recommended by the Japanese Colorectal Cancer Society (JSCCR) guideline (that is, the CME+D3 procedure) is employed, good surgical outcome is expected. With the continuous advancement of minimally invasive surgery and precise anatomization, laparoscopic radical right hemicolectomy with CME has become a standard procedure for radical resection of right colon cancer. However, due to the vascular variation of right colon, the dissection and identification of anatomies may be challenging. Improper surgical techniques may cause severe vascular injury, which may increase the surgical complexity and result in severe postoperative complications.

Yamaguchi et al. (12) found that the vascular variation is more common in the right colon, which explains the long learning curve of LRH. Henle trunk management is a challenge in the procedures of LRH and a hot topic in studies. How to safely and efficiently perform anatomical dissection of the Henle trunk and its branches is crucial for the successful surgery. In the present study, the cranial-to-caudal approach was used in 82 patients in which the gastrocolonic ligament was incised to get access into the omental sac, following the right gastroepiploic vein in a top-down manner, and then the Henle trunk and its branches were managed. In this study, this approach was superior to the traditional intermediate approach in terms of intraoperative hemorrhage, operation time and intraoperative vascular injury. Based on our results, the cranial-to-caudal approach and priority management of the Henle trunk have the following advantages. First, according to “membrane anatomy” theory (13), the cranial-to-caudal approach is more consistent with the human embryonic development. The avascular interstitial space is accessed from the pancreas head and right gastroepiploic vessel. This space is further extended to the space behind the transverse colon where the transverse colon, duodenum and pancreatic head are exposed, and the Henle trunk and middle colon vessels are dissected. This technique actually is a reverse of the torsion and fusion of the hypocotyl of mid-transverse colon during the embryonic development. The folded mesangium of right colon and transverse colon is extended to reduce the injury to the Henle trunk and the risks of vascular injury and bleeding. Meantime, it prepares for the subsequent dissection of superior mesenteric vein and regional lymph nodes. This was also accepted by Matsuda et al. (6). The middle approach usually involves the dissection of the superior mesenteric vein from bottom to the lower edge of the pancreas, the root of Henle trunk was identified and then its branches were managed. However, due to the limitation of surgical plane, excessive intraoperative mesangial traction may cause blood vessel injury and bleeding. As previously reported (14), the surface from the pancreatic head to neck is rich in blood vessel and has complicated relationship with adjacent structures. In addition, most of the blood vessels in this area are veins, which are prone to vascular injury. Due to the narrow operation view in this area, it is often difficult to control bleeding, with high risk of pancreatic injury and conversion to laparotomy due to hemorrhage. Secondly, priority management of the Henle trunk and its branches enables dissection of tumor feeding vessels in advance, which prevents and reduces the risk of tumor spread. It reflects the “NO TOUCH” principle of oncology proposed by Turnbull et al. (15). Finally, in the priority management of Henle trunk, the effort of finding and managing Henle trunk is spared when regional lymph node dissection is performed along the superior mesenteric vein in the subsequent steps. It avoids Henle trunk injury, lowers the technical threshold of surgery and shortens operation time. In this study, the short-term post-operative outcomes (such as postoperative complications, first postoperative exhaust and defecation time, postoperative drainage time, postoperative hospital stay, postoperative specimen quality evaluation, and total number of lymph nodes dissected and number of positive lymph nodes dissected) were comparable between groups. Both modalities are effective and in accordance with the principle of radical tumor treatment. The postoperative recovery and efficacy are also similar between groups.

Of course, the priority management of the Henle trunk also has certain disadvantages. It has been reported that accessing omental sac via the cephalic approach to find the Henle trunk and middle colon artery is a relatively complex technique, which tends to cause bleeding. Our experience is that accessing various spaces is not complicated, if the “membrane anatomy” concept is followed, the anatomy of mesangium and mesangial bed is well-understood, and the anatomical spaces and margins are well-identified. When CME is performed smoothly, it avoids bleeding caused by blood vessel injury. However, this technique is relatively difficult in obese patients. At the same time, if the surgeon has rich experience in gastric surgery and is familiar with the anatomical structure and variation of blood vessels from the head of pancreas to the neck of pancreas, it is still a preferred surgical technique in terms of anatomy and may not add difficulty to the surgical procedures.

There were limitations in the present study. It is a relatively new procedure, which was conducted in only one center. The sample size was still small, and there was no long-term follow-up. Thus, more studies with large sample size and long term follow up are needed to confirm our findings in the future.

In summary, the dissection and management of Henle trunk is an important step in the CME+D3 LRH for the right colon cancer. The Henle trunk management requires justified strategy and meticulous skills. We believe that the laparoscopic radical hemicolectomy combined with cranial approach for the priority management of Henle trunk and subsequent caudal intermediate is an advantageous modality for gastrointestinal surgeons with rich experience in gastric surgery, familiar with local anatomy and proficient in abdominal surgery skills. It advantages include less intraoperative blood loss, shorter operation time, and lower risk of vascular injury. It is a safe and feasible surgical procedure with favorable perioperative outcomes and has the potential for further clinical application.

## Data Availability Statement

The raw data supporting the conclusions of this article will be made available by the authors, without undue reservation.

## Ethics Statement

The studies involving human participants were reviewed and approved by Ethical Review Board of Shanghai East Hospital. Written informed consent for participation was not required for this study in accordance with the national legislation and the institutional requirements.

## Author Contributions

YY designed the study, acquired and analyzed data, and drafted the manuscript. XJ, ZZho, BL, ZZhu, QJ, and FJ acquired and analyzed data and revised the manuscript. CF contributed to the concept and design of the study, reviewed, and revised the manuscript. All authors contributed to the article and approved the submitted version.

## Funding

This work was supported by grants from the National Natural Science Foundation of China (Nos. 81773275 and 81573004), the Top-level Clinical Discipline Project of Shanghai Pudong (No. PWYgf2018-04), and the Shanghai Health and Family Planning Commission Youth Science and Technology Project (No. 202040303).

## Conflict of Interest

The authors declare that the research was conducted in the absence of any commercial or financial relationships that could be construed as a potential conflict of interest.

## Publisher's Note

All claims expressed in this article are solely those of the authors and do not necessarily represent those of their affiliated organizations, or those of the publisher, the editors and the reviewers. Any product that may be evaluated in this article, or claim that may be made by its manufacturer, is not guaranteed or endorsed by the publisher.
